# Frequent association of malignant effusions in plasmablastic lymphoma: a single-institutional experience of nine cases in Taiwan

**DOI:** 10.1007/s44313-025-00070-w

**Published:** 2025-04-07

**Authors:** Bo-Jung Chen, Yu-Ting Kuo, Sheng-Tsung Chang, Khin-Than Win, Shang-Wen Chen, Sheng-Yen Hsiao, Yin-Hsun Feng, Yen-Chuan Hsieh, Shih-Sung Chuang

**Affiliations:** 1 Department of Pathology, Shuang Ho Hospital, Taipei Medical University, New Taipei, Taiwan; 2 Department of Pathology, School of Medicine, College of Medicine, Taipei Medical University, Taipei, Taiwan; 3 Department of Radiology, Chi Mei Medical Center, Tainan, Taiwan; 4 Department of Pathology, Chi Mei Medical Center, Tainan, Taiwan; 5 Department of Nursing, National Tainan Institute of Nursing, Tainan, Taiwan; 6 Division of Hemato-Oncology, Department of Internal Medicine, Chi Mei Medical Center, Liouying, Tainan, Taiwan; 7 Division of Hemato-Oncology, Department of Internal Medicine, Chi Mei Medical Center, Tainan, Taiwan; 8 Department of Clinical Pathology, Chi Mei Medical Center, Tainan, Taiwan

**Keywords:** EBV, HIV, Malignant effusion, Multiple myeloma, Plasmablastic lymphoma

## Abstract

**Purpose:**

Plasmablastic lymphoma (PBL) is a rare, aggressive lymphoma that is characterized by terminal B-cell differentiation. In the West, PBL usually occurs in patients with immunodeficiencies, particularly those induced by human immunodeficiency virus (HIV) infection. We investigated the clinicopathological features of PBL at a single institute in Taiwan, where HIV infection is rare.

**Methods:**

This retrospective chart review identified PBL cases that were treated at a single institute in southern Taiwan between 2008 and 2024.

**Results:**

We identified nine patients (four males and five females; median age 71 years). Of the eight patients tested for HIV, only one tested positive. Pathologically, the tumors showed plasmablastic morphology and immunophenotype, and three (33%) cases tested positive for Epstein–Barr virus. Six (67%) patients presented with Stage IV disease, including five (56%) with malignant effusion. Six patients were treated with chemotherapy and the remaining three received only supportive care. During a median follow-up of 10 months, five patients died of progressive disease, two died of unrelated diseases, and two were alive with PBL relapse.

**Conclusion:**

In Taiwan, PBL constitutes a rare and aggressive clinical condition and is frequently associated with malignant effusion. In contrast to Western patients, the PBL in most patients from Taiwan was unrelated to HIV infection.

## Introduction

Plasmablastic lymphoma (PBL) is a rare, aggressive large B-cell lymphoma that is characterized by large atypical B cells with plasmablastic or immunoblastic morphology and a terminal B-cell differentiation phenotype – that is, the expression of plasma cell-related antigens (CD38, CD138, MUM1, PRDM1 [BLIMP1], and/or XBP1) but not common B-cell antigens (CD20 and PAX5). PBL frequently occurs in patients with immune deficiency or dysregulation, such as human immunodeficiency virus (HIV) infection, and in patients with prior organ transplantation, autoimmune diseases, and anti-CD19 CART therapy [1–5]. Based on a single-institution study of 25 cases, Morscio et al. reported that PBL could originate in several different clinical settings, including in patients with acquired immunodeficiency syndrome (50%), immunocompetent individuals (35%), and transplant recipients (14%) [2]. Furthermore, PBL may represent a high-grade transformation from mature B-cell lymphomas, such as follicular lymphoma and chronic lymphocytic leukemia/small lymphocytic lymphoma [6].

PBL usually involves extranodal sites, such as the nasal/oral cavity, digestive system, bone, soft tissues, and skin, but rarely involves malignant effusions [5]. The identification of rare cases of PBL with malignant effusion in routine clinical practice motivated this retrospective investigation of PBL. This case series was developed to better characterize the association between malignant effusion and other clinicopathological features of PBL in Taiwan, which has low HIV infection rates. 

## Materials and methods

This retrospective chart review identified PBL cases that presented between January 2008 and December 2024 at a medical center, a regional hospital, and a community hospital, all run by the Chi-Mei Medical Group, which comprises a total of 2,532 beds. PBL was diagnosed from clinical, laboratory, and imaging findings, particularly with the exclusion of plasma cell myeloma/multiple myeloma (MM), in accordance with the diagnostic criteria in the WHO Classification of Haematolymphoid Tumors, 5th Edition (WHO-HAEM5) [5]. Lymphoplasmacytic lymphoma (LPL), which comprises small B cells, plasmacytoid lymphocytes, and mature plasma cells, constitutes a differential diagnosis for PBL. LPL usually involves the bone marrow (BM) and, sometimes, the lymph nodes; however, LPL rarely extends to extranodal sites. In contrast to the small nuclear size and low Ki67 labeling index of neoplastic cells in LPL, the PBL samples in this case series comprised large, blastic neoplastic cells with vesicular nuclei and a high labeling index on Ki67 immunostaining.

## Results

**Fig. 1:**
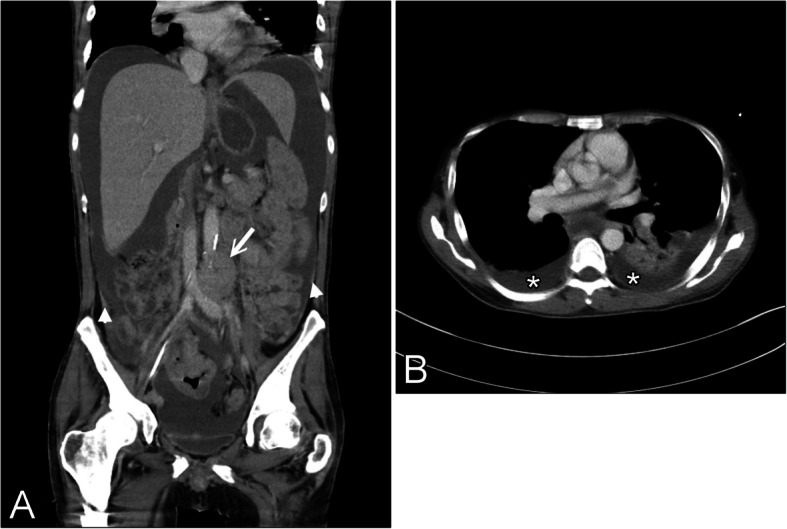
A 41-year-old, HIV-infected male patient (Patient no. 9) underwent imaging studies after diagnosis. Post-contrast-enhanced coronal CT images of the abdomen (**A**) and axial CT images of the lower chest (**B**) demonstrate a large volume of ascites and bilateral pleural effusions (*). An enlarged para-aortic lymph node (arrow) and peritoneal nodular thickening (arrowheads) are observed. Partial collapse of the left lower lobe of the lung is also noted

Among the 1,921 cases of malignant lymphoid neoplasms that were recorded during the study period, we identified nine patients (0.47%) with PBL. These patients included four males and five females (age, median [range] 71 [35–92] years). Table 1 lists the relevant clinical characteristics of these patients. Patient nos. 1–4 were included in our previous multi-institutional genetic study on PBL [7]. Patient no. 3 was previously reported to have a double-hit PBL with malignant effusion [8]. According to the Lugano Classification, three patients had early-stage PBL (IE, II, and IIE) whereas six had Stage IV PBL. Among those with Stage IV PBL, five (patient nos. 1, 3, 6, 8, and 9) demonstrated involvement of body cavities, including lymphomatous pleural effusion, which was noted at disease presentation in four patients, of whom three (nos. 3, 8, and 9) had concurrent involvement of the peritoneal cavity/ascites. The last patient (no. 9) had multi-site lymphadenopathy in the thoracic and abdominal cavities, bilateral pleural effusions, and peritoneal “carcinomatosis” with massive ascites that were detected on CT scans (Fig. 1). Although there was no cytological confirmation, lymphomatous pleural effusion and malignant ascites were suspected.

**Table 1:** Clinical features of PBL in a single-institution case series

Case no./Sex/Age	Initial diagnostic specimen(s)	Involved organs/sites	HIV status	Immuno- deficiency	Stage	IPI score	Treatment	FU (mo.)	Survey for MM^d^
1/F/71	Neck LN	Generalized LAP, right pleural seeding with lymphomatous PE	Negative	No	IV	3	CEOP	DOD (64)	Negative
2/M/67	Neck LN	LN, neck	Negative	No	II	1	DA-EPOCH, CODOX-M, IVAC, ICE, palliative RT	DOD (10)	Negative
3/F/35	Neck LN/soft tissue	LN, soft tissue, spine, PB, BM, lymphomatous PE and ascites	Negative	No	IV	4	CHOP × 1	DOD (0.5)	Negative
4/F/75	Gingiva and mandible	Gingival mass invading the underlying mandible	Negative	No	IE	1	Reduced dose CHOP × 3, RT 4500 cGy	DOUD (16)^a^	Negative
5/M/92	Gingiva	Gingiva, right lower, regional LNs	Negative	No	IIE	1	Supportive care	DOUD (23)^b^	Negative
6/F/41	Neck LN, NP, breast	NP, right breast, generalized LAP, PE, BM, bones	Negative	No	IV	3	EPOCH × 6. HD Ara-C, IT MTX, Daratumum	AWD (10)^c^	Negative
7/F/73	NP, skin	Disseminated. Generalized LAP, NP, generalized cutaneous lesions, bones	Negative	No	IV	4	CHOP × 3 plus Bortezomib	AWD (6)	Negative
8/M/74	UB, skin, PE	Generalized LAP, pleural and peritoneal cavities, abdominal and pelvic organs	ND	No	IV	5	Supportive care	DOD (0.1)	ND^e^
9/M/41	BM	Peritoneum, generalized LAP, lymphomatous PE and ascites	Positive	Yes	IV	4	Supportive care	DOD (0.6)	Negative

*Abbreviations: AWD* alive with disease, *BM* bone marrow, *DOD* died of disease, *DOUD* died of unrelated disease, *FU (mo.)* follow-up status in months, *HD Ara-C* high-dose cytarabine, *IT-MTX* intrathecal methotrexate, *LAP* lymphadenopathy, *LN* lymph node, *MM* multiple myeloma, *ND* not done, *NP* nasopharynx, *PB* peripheral blood, *PE* pleural effusion, *RT* radiotherapy, *UB* urinary bladder

^a^Patient No. 4 died of metastatic urothelial carcinoma in 16 months while her PBL was in complete remission

^b^Patient No. 5 died of pneumonia with other co-morbidities including diabetes mellitus, grade 4 chronic kidney disease, and chronic obstructive pulmonary disease

^c^Patient No. 6 achieved complete remission after six course of EPOCH chemotherapy, but relapsed in seven months with tumor in the spine, intradural tumor seeding and involvement of cerebro-spinal fluid (confirmed by cytology and flow cytometric immunophenotyping)

^d^Survey for MM included the detection of M protein by immunoelectrophoresis, CRAB (hypercalcemia, impaired renal function with elevated creatinine level, anemia, and lytic bony lesions), serum immunoglobulins and free light chains

^e^The clinical course of Patient No. 8 was rapid, defying investigation for MM. The only evidence of absence of osteolytic lesions was from X-ray

The HIV assay was performed in eight patients (nos. 1–7 and 9) and was positive in only one patient (no. 9). MM was excluded in eight patients (nos. 1–7 and 9; Table 2). The International Prognostic Index (IPI) scores were 1 (*n* = 3), 3 (*n* = 2), 4 (*n* = 3), and 5 (*n* = 1). Six patients received various chemotherapy regimens, including two who received additional radiotherapy. The remaining three patients were managed supportively because of either old age (no. 5) or a rapidly progressive course (nos. 8 and 9). The median follow-up duration was 10 (range, 0.1–64) months. At the last follow-up, five patients died of the disease (survival duration: 0.1, 0.5, 0.6, 10, and 64 months, respectively), two died of unrelated diseases, and two were alive with relapsed diseases (3 and 7 months, respectively). Patient no. 4 died of metastatic urothelial carcinoma within 16 months when her PBL was in complete remission. Patient no. 5, the oldest patient (aged 94 years) in this cohort, died of pneumonia with other comorbidities at 23 months, with an indeterminate PBL status.

**Table 2:** The results of investigations undertaken for multiple myeloma

Case no./Sex/Age	Monoclonal protein (by IEP)	Ig (mg/dL)	Hypercalcemia (calcium, mg/dL)	Renal insufficiency (creatinine, mg/dL)	Anemia (Hb, g/dL)	Lytic bone lesions (methods of detection)	Serum free light chains (mg/dL)	Clonal PCs in BM
1/F/71	NA	NA	NA	Absent (1.77)	Absent	Absent^a^ (bone scan)	NA	Absent
2/M/67	NA	NA	Absent	Absent (1.13)	Absent	Absent (CT)	κ: 22.8, λ: 17.0 ratio: 1.34	NA
3/F/35	NA	NA	Absent (8.2)	Absent (0.98)	Absent	Absent (X-ray)	NA	Absent
4/F/75	NA	IgG 988 IgA 212 IgM 45	Absent (8.8)	Absent (0.94)	Absent	Absent (CT, PET-CT, and MRI)	κ: 61.84, λ: 33.87 ratio: 1.83	Absent
5/M/92	Absent	IgG 1395 IgA 178 IgM 58	Absent (9.4)	Absent (1.72)	Mild (12.5)	Absent (PET-CT)	κ: 74.94, λ: 44.79 ratio: 1.67	Absent
6/F/41	Absent	IgG 1092 IgA 234 IgM 82	Absent (9.3)	Absent (0.60)	Mild (10.8)	Absent (CT and MRI)	κ: 8.2 λ: 15.9 ratio: 0.52	Absent
7/F/73	Absent	IgG 497 IgA 66 IgM 27	Absent (9.3)	Absent (0.77)	Absent	Absent (CT and MRI)	κ: 8.7 λ: 8.1 ratio: 1.07	Absent
8/M/74	NA	NA	Absent (8.4)	Present^b^ (1.85—> 5.15)	Mild (11.2)	Absent (X-ray)	NA	NA
9/M/41	NA	NA	Absent (9.2)	Absent (0.79)	Mild (11.7)	Absent (X-ray and CT)	NA	Absent

Reference ranges in our institute: calcium, 8.4–10.2 mg/dL; creatinine, 0.57–1.11 mg/dL; IgA, 65–421 mg/dL; IgG, 970–2640 mg/dL; IgM, 33–293 mg/dL

*Abbreviations: BM* bone marrow, *Hb* haemoglobin, *IEP* immunoelectrophoresis, *Ig* immunoglobulin, *MM* multiple myeloma, *NA* not available, *PC* plasma cell

^a^Case no. 1. Bone scan 6 months after the diagnosis of plasmablastic lymphoma revealed insufficiency fracture of the pelvis and lumbar spine L2 compression fracture, without osteolytic lesions

^b^The renal insufficiency with rapidly increasing serum creatinine levels in Case no. 8 was due to lymphomatous invasion leading to bilateral hydronephrosis, obstructive uropathy, and acute kidney injury

**Fig. 2:**
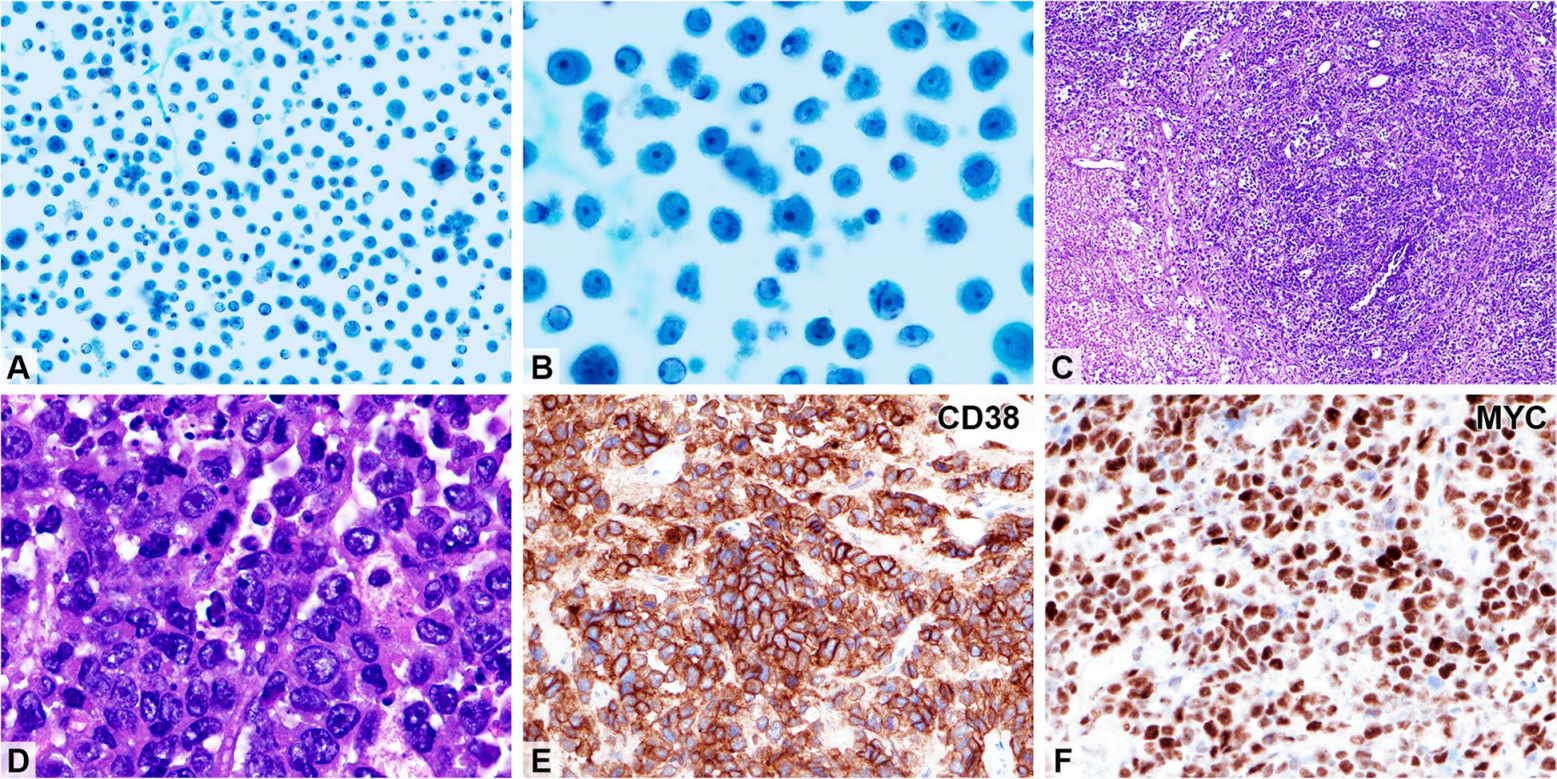
A representative case of plasmablastic lymphoma (Patient no. 8). The patient presented with disseminated disease and pleural effusion. Effusion cytology from cytospun smear shows numerous discohesive tumor cells with vesicular nuclei and slightly irregular nuclear contours and the majority with a single prominent nucleolus (**A** × 400 and **B** × 1000; Papanicolaou stain). **C**–**F**, Trans-urethral resection specimen of the urinary bladder shows a diffuse lymphomatous infiltration with focal tumor necrosis (**C**; HE stain × 100). The tumor cells are large with vesicular nuclei, prominent nucleoli, and a moderate amount of eosinophilic to amphophilic cytoplasm (**D**; HE stain × 1,000). The tumor cells diffusely express CD38 (**E**; immunohistochemical stain × 400) and MYC (**F**; × 400), but not CD20 (not shown), and tested negative for EBV on in situ hybridization (not shown)

As listed in Table 3, the initial diagnostic specimens were obtained from the lymph nodes (*n* = 3), oral cavity (*n* = 2), nasopharynx (*n* = 2), subcutis (*n* = 1), and bone marrow (*n* = 1). The affected nodes and nasopharynx were completely effaced by diffuse sheets of large atypical lymphocytes with round nuclei, prominent nucleoli (usually a single nucleolus), and eosinophilic to amphophilic cytoplasm (Fig. 2). Immunohistochemical findings are presented in Table 3. All cases tested negative for the B-cell marker CD20 and PAX5, except one patient (no. 3) in whom the tumor cells were positive for the plasma cell-related markers CD138 and MUM1. MYC was expressed in 78% (7/9) of the cases, which is consistent with the literature [5, 7, 9]. EBV), as determined by in situ hybridization, was positive in three cases (33%).

**Table 3:** Immunophenotype including EBER

Case no	Bx. site	CD19	CD20	CD79a	CD138	PAX5	MUM1	MYC	HHV8	EBER	Others
1	LN, neck	ND	Neg	Pos	Pos	Neg	Pos	Pos	Neg	Neg	
2	LN, neck	Neg	Neg	Neg	Pos	Neg	Pos	Pos	Neg	Neg	
3	LN, neck	Pos	Neg	Pos	Pos	Pos	Pos	Pos	Neg	Neg	
4	Oral mucosa	ND	Neg	Pos	Neg	Neg	Pos	Pos	Neg	Pos (partial)	
5	Oral mucosa	Neg	Neg	ND	Pos (partial)	Neg	Pos	Neg	Neg	Pos	
6	NP	Neg	Neg	Neg	Neg	Neg	Neg	Pos	Neg	Neg	CD38 ( +), CD45( +), CD56 ( +), CD117(-), Cyclin D1(-)
7	NP and skin	Pos	Neg	ND	Pos	Neg	Pos	Neg	Neg	Pos	
8	Subcutis	Neg	Neg	Neg	Pos (partial)	Neg	Pos	Pos	Neg	Neg	CD38 ( +)
9	BM	Neg	Neg	Neg	Pos	Neg	Pos	Pos	Neg	Neg	CD56 (-), CD117 (-), Cyclin D1 (+ P)
Pos rate		29% (2/7)	0% (0/9)	43% (3/7)	78% (7/9)	11% (1/9)	89% (8/9)	78% (7/9)	0% (0/9)	33% (3/9)	

*Abbreviations: BM* bone marrow, *Bx.* biopsy, *LN* lymph node, *ND* not done, *Neg* negative, *NP* nasopharynx, *+ P*, partially positive, *Pos* positive

## Discussion

In the West, PBL usually occurs in patients with HIV infection; however, in Taiwan, the relative frequency of HIV infection in patients with PBL is low. Recently, we reported the only series of PBL cases in Taiwan, with 26 cases enrolled from eight hospitals in collaboration with the Taiwan Hematopathology Study Group [7]. Among these patients, only five (5/18; 28%) tested positive for HIV. In the current study, only one patient (no. 9) was infected with HIV, whereas patients nos. 1–7 were all negative for HIV. Patient no. 8 was not tested for HIV infection because of its aggressive clinical course. However, he was probably not infected with HIV because older people in Taiwan are usually not HIV-infected. To the best of our knowledge, only one other case of HIV-related PBL has been reported in Taiwan [10].

In a study of 50 cases, Colomo et al. reported that diffuse large B-cell lymphomas with plasmablastic differentiation represented a heterogeneous group of entities, including HIV-related PBL of the oral mucosal type (*n* = 23), PBL with plasmacytic differentiation (*n* = 16), secondary involvement of plasmablastic MM (*n* = 9), and three others [11]. Their study underscored the challenges and importance of differentiating PBL from plasmablastic MM, given the different treatment modalities for these two diseases. Patient no. 7 presented to an otolaryngologist with neck masses, and a nasopharyngeal biopsy was performed to exclude nasopharyngeal carcinoma, which is prevalent in Taiwan. Based on the biopsy, the patient was initially diagnosed with Epstein-Barr virus-positive plasmacytoma, as the infiltrating plasma cells were atypical plasma cells with abundant cytoplasm but without blastic or large nuclei. However, a subsequent cutaneous biopsy revealed sheets of large tumor cells with blastic morphology and a plasmacytic phenotype. Furthermore, the investigations for MM were negative. Therefore, the diagnosis was revised to PBL, specifically a PBL with plasmacytic differentiation, which constitutes the 2nd subgroup described by Colomo et al. [11].

Plasmacytic neoplasms, including plasmacytoma and MM, present major diagnostic challenges in PBL. As highlighted in a previous review, this distinction relies mainly on the detection of monoclonal paraproteins and MM-related end-organ damage, such as CRAB (hypercalcemia, renal insufficiency with elevated creatinine levels, anemia, and lytic bone lesions) [4, 5]. In our cohort, the first seven patients tested negative for any evidence of MM after a systemic survey. The 8th patient was not surveyed because of his aggressive clinical course; however, his serum calcium level was normal and there were no osteolytic lesions on radiography, excluding MM. The 9th patient, an HIV-infected male, was not surveyed for MM because of his aggressive course. He had normal serum levels of calcium and creatinine and no evidence of bony lesions on radiography and CT scans, making MM unlikely.

According to the WHO-HAEM5, PBL usually involves extranodal sites, such as the nasal/oral cavity (~ 50%), digestive system (~ 20%), bone and soft tissues (~ 15%), and skin (~ 5%) [5]. There are only a few reports of patients with PBL presenting with malignant effusion [8, 12–16]. In routine diagnostic cytopathology services, malignant effusion is usually caused by an underlying carcinoma, such as lung cancer in the pleural effusion or gastrointestinal or gynecological cancers with malignant ascites, but not by lymphoma [17, 18]. In our previous study of 43 cases of lymphomatous effusion at a tertiary center in Taiwan, the lymphomatous effusion was either primary (16%) or secondary (84%) [19]. Cytological examination are mandatory in patients with pleural/peritoneal effusions. Cellular atypia and clinical history are the most important factors for triaging lymphocyte-rich effusion specimens in ancillary studies to reach a correct diagnosis [20]. In the current study, five of the nine patients presented with malignant effusion. Our findings suggest that malignant effusion may not be rare in PBL, and this contradicts current perceptions. We suspect that one of the reasons for this underreporting may be underdiagnosis, either because the body fluids are not routinely submitted for cytological examination or because the laboratory staff are not familiar with the cytological features/ancillary examinations for lymphocyte-rich effusions. Thus, whether malignant effusion is a significant prognostic factor for PBL warrants a large-scale systemic study.

In the first report of HIV-related PBL, 60% (9/15) of the cases were EBV-related [1]. The lower EBV association rate (33%) in our cohort may be attributable to the low rate of immunodeficiency, including HIV infection. Five of our patients were older than 70 years; therefore, we suspect that immunosenescence might be a major underlying cause of non-HIV-related PBL in Taiwan. The prognosis of PBL is poor, with a median overall survival of 6–32 months [5]. In a recent review, age ≥ 60 years, advanced stage, and high intermediate and high IPI scores were found to be poor prognostic factors [21].

In this largest case series of PBL from a single institution in Taiwan, we found that PBL frequently involves the body cavities, particularly malignant pleural effusion. In addition, PBL is aggressive, usually HIV-unrelated, and might be related to immunosenescence.

## Data Availability

No datasets were generated or analysed during the current study.
